# DUAL DECLINE IN MEMORY AND GAIT UNIQUELY IDENTIFIES OLDER PERSONS AT HIGH RISK OF DEMENTIA

**DOI:** 10.1093/geroni/igz038.2173

**Published:** 2019-11-08

**Authors:** Qu Tian, Susan Resnick, Michelle Mielke, Kristine Yaffe, Caterina Rosano, Eleanor M Simonsick, Stephanie Studenski, Luigi Ferrucci

**Affiliations:** 1 National Institute on Aging, Baltimore, Maryland, United States; 2 Mayo Clinic College of Medicine, Rochester, Minnesota, United States; 3 UCSF School of Medicine, San Francisco, California, United States; 4 University of Pittsburgh, PIttsburgh, Pennsylvania, United States; 5 Longitudinal Studies Section, Intramural Research Program, National Institute on Aging, Baltimore, Maryland, United States; 6 NIA, Longitudinal Studies Section, Baltimore, Maryland, United States; 7 Translational Gerontology Branch, Intramural Research Program, National Institute on Aging, National Institutes of Health, Baltimore, Maryland, United States

## Abstract

Recent study has shown that incident dementia risk is higher among older persons who decline in both cognition and gait. This study assesses whether this relationship exists in multiple other aging populations. Data are from the Baltimore Longitudinal Study of Aging (n=662, 51%women,22%blacks), Health, Aging and Body Composition Study (n=746, 51%women,44%blacks), and Mayo Clinic Study of Aging (n=2771, 48%women,0.3%blacks). Participants were at least 60, initially free of cognitive impairment, dementia, and dismobility (gait speed≤0.6m/s), with repeated measures of verbal memory and gait speed before dementia diagnosis (average follow-up 5.8-12.1 years). Within each cohort, participants were divided into four groups: memory decliners alone, gait decliners alone, dual decliners, or neither (healthy agers). Gait speed decline was defined as a loss of ≥0.05m/sec/year; memory decline was defined as cohort specific bottom slope tertile. Incident dementia risk was examined by Cox regression with healthy agers as reference, adjusted for sex, race, baseline age, gait speed and memory. Across studies, incident dementia ranged from 3% to 17%. Compared to healthy agers, memory decliners alone had 3.4 to 4.3 times higher risk for developing dementia (p<0.01). Gait decliners alone had 2.1-5.6 times higher risk for dementia (p<0.05). Dual decliners had 7.6-10.8 times the risk (p<0.001). Dual decline signifies a more rapid progression to dementia. These consistent findings suggest that dual decliners might be a potentially valuable target group for both preventive interventions and mechanistic studies. Whether dual decline identifies a particular subtype or multiple subtypes of dementia remains to be investigated.

